# Bone Marrow Deficiency of MCPIP1 Results in Severe Multi-Organ Inflammation but Diminishes Atherogenesis in Hyperlipidemic Mice

**DOI:** 10.1371/journal.pone.0080089

**Published:** 2013-11-06

**Authors:** Fang Yu, Fen Du, Yuzhen Wang, Shengping Huang, Ruidong Miao, Amy S. Major, E. Angela Murphy, Mingui Fu, Daping Fan

**Affiliations:** 1 Department of Nutrition and Food Hygiene, the Fourth Military Medical University, Xi’an, Shaanxi, China; 2 Department of Cell Biology and Anatomy, University of South Carolina School of Medicine, Columbia, South Carolina, United States of America; 3 Department of Biochemistry and molecular Biology, School of Basic Medicine, Wuhan University, Wuhan, China; 4 Shock/Trauma Research Center & Department of Basic Medical Science, School of Medicine, University of Missouri Kansas City, Kansas City, Missouri, United States of America; 5 Department of Medicine, Division of Cardiovascular Medicine, Vanderbilt University Medical Center, Nashville, Tennessee, United State of America; 6 Department of Pathology, Microbiology and Immunology, University of South Carolina School of Medicine, Columbia, South Carolina, United States of America; Morehouse School of Medicine, United States of America

## Abstract

**Objective:**

MCPIP1 is a newly identified protein that profoundly impacts immunity and inflammation. We aim to test if MCPIP1 deficiency in hematopoietic cells results in systemic inflammation and accelerates atherogenesis in mice.

**Approach and Results:**

After lethally irradiated, LDLR^−/−^ mice were transplanted with bone marrow cells from either wild-type or MCPIP1^−/−^ mice. These chimeric mice were fed a western-type diet for 7 weeks. We found that bone marrow MCPIP1^−/−^ mice displayed a phenotype similar to that of whole body MCPIP1^−/−^ mice, with severe systemic and multi-organ inflammation. However, MCPIP1^−/−^ bone marrow recipients developed >10-fold less atherosclerotic lesions in the proximal aorta than WT bone marrow recipients, and essentially no lesions in *en face* aorta. The diminishment in atherosclerosis in bone marrow MCPIP1^−/−^ mice may be partially attributed to the slight decrease in their plasma lipids. Flow cytometric analysis of splenocytes showed that bone marrow MCPIP1^−/−^ mice contained reduced numbers of T cells and B cells, but increased numbers of regulatory T cells, Th17 cells, CD11b+/Gr1+ cells and CD11b+/Ly6C^low^ cells. This overall anti-atherogenic leukocyte profile may also contribute to the reduced atherogenesis. We also examined the cholesterol efflux capability of MCPIP1 deficient macrophages, and found that MCPIP1deficiency increased cholesterol efflux to apoAI and HDL, due to increased protein levels of ABCA1 and ABCG1.

**Conclusions:**

Hematopoietic deficiency of MCPIP1 resulted in severe systemic and multi-organ inflammation but paradoxically diminished atherogenesis in mice. The reduced atheroegensis may be explained by the decreased plasma cholesterol levels, the anti-atherogenic leukocyte profile, as well as enhanced cholesterol efflux capability. This study suggests that, while atherosclerosis is a chronic inflammatory disease, the mechanisms underlying atherogenesis-associated inflammation in arterial wall versus the inflammation in solid organs may be substantially different.

## Introduction

Monocyte chemotactic protein-induced protein 1 (MCPIP1), also known as ZC3H12A, is a novel CCCH-zinc finger-containing protein [1], [2]. It can be induced in macrophages upon stimulation with proinflammatory molecules such as TNFα, MCP-1, IL-1β and LPS [3]. It exerts negative feedback to inhibit LPS-induced TNFα and iNOS promoter activation in macrophages through deubiquitinating TRAF proteins [1], or to directly control the mRNA stability of a set of inflammatory genes including IL-6 [4], IL-1β [5] and IL-2 [6] in immune cells, and particularly in macrophages. It also fine-tunes inflammatory responses by modulating microRNA maturation and function [7]. Therefore, it is a potent negative regulator in immune cell activation and inflammatory responses, playing a crucial role in hemostasis maintenance of immune system function. MCPIP1 deficient mice display a complex phenotype, including growth retardation, severe anemia, and severe inflammatory response; most mice die within 12 weeks of age due to severe systemic inflammation and multiple organ functional failure [4]. Although it has been suggested that hematopoietic cell deficiency of MCPIP1 may transfer some of the phenotype of MCPIP1 knockout [4], the detailed phenotype of the bone marrow MCPIP1 deficient mice has not yet been characterized.

Atherosclerosis is a chronic inflammatory disease; all immune components participate in atherogenesis, with the macrophage inflammatory response to oxidized LDL serving as an important initial event [8]–[11]. It is thought that the interplay between traditional risk factors, such as LDL cholesterol (hyperlipidemia) and angiotensin II (hypertension), and the inflammatory response machinery, can orchestrate the interaction between arterial wall cells (endothelial cells and smooth muscle cells) and immune cells (mainly monocytes/macrophages, T and B lymphocytes), leading to pathogenesis of the disease [12], [13]. Despite its well appreciated involvement in atherogenesis, a causative role of inflammation in this disease has yet to be established. And to date, there are no documented anti-inflammatory drugs that have been proven beneficial in atherosclerotic vascular disease patients. Currently there are two clinical trials to directly test the efficacy of anti-inflammatory therapy in atherosclerosis; one is the Canakinumab Anti-Thrombosis Outcome Study (CANTOS) that is testing the cardiovascular event reducing effects of IL-1β neutralizing antibody [14], and the other is the Cardiovascular Inflammation Reduction Trial (CRIT) that is examining whether low-dose methotrexate treatment yields beneficial effects to cardiovascular patients [15]. These two trials are expected to prove or disprove the inflammatory hypothesis of atherogenesis.

Because of the important role of MCPIP1 as an inflammation modulator and the inflammatory nature of atherosclerosis, we expect that MCPIP1 deficient mice will be an excellent mouse model to validate the inflammatory hypothesis of atherogenesis and may also serve as a model to test the anti-atherogenic efficacy of anti-inflammatory agents. However, the premature death of the MCPIP1 deficient mice makes it difficult to cross these mice to either apoE^−/−^ or LDLR^−/−^ mice to generate an atherosclerosis-prone mouse model. Therefore, we used a bone marrow transplantation approach to investigate the effects of bone marrow cell MCPIP1 deficiency on atherosclerosis development in LDLR^−/−^ mice fed a western-type diet. Interestingly, we found that even though bone marrow deficiency in MCPIP1 transferred the major phenotype of whole body MCPIP1 deficiency, including severe systemic inflammation, surprisingly these mice developed much less atherosclerosis than those with WT bone marrow. While the slightly reduced plasma cholesterol levels and an altered leukocyte profile in the bone marrow MCPIP1 deficient mice may at least partially contribute to the reduced atherogenesis, the paradoxical dissociation between systemic inflammation and atherogenesis in this chimeric mouse model may provide a novel platform for further evaluation of the role of inflammation in atherogenesis.

## Materials and Methods

### Mice

Wild type (WT) and LDLR^−/−^ mice (C57BL/6J background) were obtained from the Jackson Laboratory (Bar Harbor, Maine). MCPIP1^−/−^ mice were also on a C57BL/6 background [3].

### Ethics statement

All animal procedures were approved by the Institutional Animal Care and Use Committee (IACUC) of the University of South Carolina with an Animal Usage Protocol (AUP) number 2077-100617-050712.

### Bone marrow transplantation

Bone marrow cells were harvested by flushing both femurs and tibias from donor mice (WT or MCPIP1^−/−^ mice) with sterile RPMI medium 1640 (Invitrogen Life Technologies, Grand Island, NY) containing 2% fetal bovine serum (FBS, Invitrogen) and heparin (5 units/ml, Sigma, St. Louis, MO). The cell suspension was centrifuged for 5 min at 340× g and re-suspended in ice-cold phosphate buffer saline (PBS). Recipient LDLR^−/−^ female mice (six-week-old) were lethally irradiated (9 Gy) by a cesium gamma source. Six hours later, 5×10^6^ donor bone marrow cells were injected into the retro-orbital venous plexus of irradiated LDLR^−/−^ mice. Four weeks after bone marrow transplantation, mice were started on either a chow diet (0.025% cholesterol) or a high-fat diet (HFD, 21% anhydrous milkfat, 34% sucrose, and a total of 0.2% cholesterol) (Harlan Laboratories, Indianapolis, IN) for a duration of 7 weeks.

### VetScan hematology analysis

Blood was drawn into heparinized tubes via retro-orbital puncture. Blood samples were run on the VetScan HMT hematology analyzer (Abaxis, Union City, CA). The following parameters were measured: total white blood cells (WBC), total red blood cells (RBC), hemoglobin, platelet number, lymphocytes, mean cell volume (MCV), mean corpuscular hemoglobin (MCH), mean corpuscular hemoglobin concentration (MCHC), hematocrit (Hct), red cell distribution width (RDW), platelet distribution width (PDW), and mean platelet volume (MPV).

### Blood smear preparation and Giemsa staining

One drop of fresh blood was placed on a coverslip and then quickly dispersed in a monolayer without disrupting cells. The smears were rapidly dried to avoid contraction or artifacts and were stained with Wright-Giemsa stain (Polysciences, Warrington, PA) according to the manufacturer’s instructions.

### Plasma lipid analysis

Blood was obtained from mice following an overnight fast and the plasma was obtained by centrifugation of blood at 4000 g for 20 minutes. Plasma cholesterol and triglyceride levels were measured by enzymatic colorimetric assays with Cholesterol Reagent and Triglycerides GPO reagent kits (Raichem, San Diego, CA). Plasma cholesterol lipoprotein profiles were determined using a fast-performance liquid chromatography (FPLC) system (AKTA purifier, GE Healthcare Biosciences, Pittsburgh, PA) equipped with a Superose 6 10/300 GL column (GE Healthcare). Pooled mouse plasma (100 µl) was loaded onto the column, and eluted at a constant flow rate of 0.5 ml/min with 1 mM sodium EDTA and 0.15 M NaCI. Fractions of 0.5 ml were collected and cholesterol concentration from each fraction was measured.

### Total IgG and anti-oxLDL IgG concentrations in plasma

Total IgG and anti-oxLDL IgG concentration were determined by enzyme-linked immunosorbent assay in plasma samples as previously described [16]. Serum IgG was measured by coating MaxiSorp ELISA plates (Nalge Nunc, Rochester, NY) with 0.5 ug/mL IgG heavy and light chain (Southern Biotech, Birmingham, AL). Plates were blocked 1h with 10% FBS in 1X PBS and serum samples or mouse reference serum (Bethyl Laboratories, Montgomery, TX) were incubated overnight at 4°C. Detection antibody [0.5 ug/ml biotin-conjugated anti-IgM, -IgG1, -IgG2c (Southern Biotech, Birmingham AL), or anti-IgG HRP-conjugated antibody (1:2500) (Promega, Madison, WI)] was incubated 2 hours at RT. Streptavidin-HRP (1:2500)(Sigma, St. Louis, MO) was incubated in IgM, IgG1, or IgG2c wells for 1 hour at RT before plates were developed with OptEIA TMB Substrate (BD Biosciences, San Diego, CA). OxLDL-specific IgG was measured in a similar manner with the exception of coating plates with oxLDL (10 mg/ml) in PBS overnight at 4°C.

### Flow cytometrical analysis

Splenocytes were obtained by gentle disruption of the organs in PBS/2% FBS followed by depletion of red blood cells using red blood cell lysing buffer (Sigma). Thymus glands and lymph nodes were digested with collagenase II and DNase (Washington, Lakewood, NJ) for 30 min at 37°C. Cells were incubated with CD16/32 antibody (eBioscience, San Diego, CA) in staining buffer (PBS/2% FBS) to block nonspecific binding, followed by staining with the cell surface antibodies at 4°C for 30 min. For intracellular cytokine staining, the cells were seeded onto 24-well plates and stimulated with 100 ng/ml Phorbol myristate acetate (PMA, Sigma), 1 µg/ml Ionomycin (Sigma) and Golgi Stop (1.5 µl in 2 ml culture medium) (BD Biosciences, San Jose, CA) for 4 h. Cells were fixed, permeabilized and stained with the following primary antibodies: PE-anti-CD11b, FITC-anti-Ly6C, FITC-anti-Ly6G, PE-anti-CD3, FITC-anti-CD4, FITC-anti-CD8a, FITC-anti-CD19, FITC-anti-Gr-1, PE-anti-IL17, PE-anti-FOXP3. All these antibodies were purchased from eBioscience. Data were acquired on a Cytomics FC 500 flow cytometer and were further analyzed with CXP software version 2.2 (Beckman coulter, Brea CA). Data were collected for 10,000 live events per sample. Leukocyte subpopulation numbers were calculated as total leukocytes multiplied by percent cells within the selected population gated by flow cytometry analysis.

### Plasma cytokine concentration measurement by ELISA

A standard sandwich ELISA was used to measure circulating TNF-α and IL-6 concentration according to the manufacturer’s instructions (R&D Systems, Minneapolis, MN). Briefly, 96-well ELISA plates were coated with 100 µl of TNF-α or IL-6 capture antibody (2 µg/ml) and incubated overnight at 4°C. The cells were then incubated with 300 µl of blocking solution (1% BSA, 5% sucrose, and 0.05% NaN3) for 1 h at room temperature. Next, 100 µl of samples were loaded to each well and incubated for 2 h at room temperature. After washing three times, the plates were successively incubated with biotinylated anti-mouse TNF-α or IL-6 (250 ng/ml), 1 µg/ml horseradish peroxidase streptavidin and substrate solution. The reaction was stopped by adding 50 µl of 1M H_2_SO_4_ solution. Recombinant mouse TNF-α or IL-6 was used to generate a linear standard curve. Optical density was determined with a SpectraMax M5 microplate reader (Molecular Devices, Sunnyvale, CA) at 450 nm. TNF-α and IL-6 concentrations are expressed as the average of two different dilutions for each sample.

### Histology

Tissues were dissected and fixed in 4% paraformaldehyde and placed in a 30% sucrose solution at 4°C overnight. Tissues were frozen in Tissue Tek OCT compound (Sakura Finetek) and sliced into 5-µm sections. Cryosections were stained with hematoxylin (GeneTex, cat# GTX73341) and eosin (Sigma-aldrich, cat# 861006-10G), and viewed with a Nikon Eclipse E600 microscope (Nikon, Inc., Melville, NY).

### Atherosclerosis analysis

The chest and peritoneal cavity were opened and the circulatory system was perfused with PBS via the left ventricle. The Aortic root was embedded in OCT medium and frozen in –20 °C immediately following excision from the aorta. The aorta was dissected and fixed in 10% neutral buffered formalin at room temperature overnight and then kept in PBS before removing fatty tissue. Successive 10-µm transversal sections of aortic sinus were obtained from the aorta root on a cryostat. The lesion area of the frozen sections was detected by H&E staining. The *en face* aortas were stained with Oil Red O. The plaque areas were analyzed by ImageJ.

### Macrophage cholesterol efflux

Bone marrow cells obtained from WT or MCPIP1^−/−^ mice were induced to differentiate into macrophages as previously described [17]. Attached mouse macrophages in 24-well plates were cultured in DMEM for 24 h followed by cholesterol-loading with 100 μg/ml acetylated LDL (ac-LDL) incorporated with ^3^H-cholesterol for 3 d. Then efflux medium (either FBS-free DMEM or DMEM containing 20 µg/ml human apoAI or 50 µg/ml human HDL) was added to initiate cholesterol efflux for 24 h. ^3^H radioactivity in efflux medium and cells were counted using a Beckman LS6500 liquid scintillation counter (Beckman Coulter, Indianapolis, IN). Cholesterol efflux rate was calculated as the percentage of the radioactivity counts in the medium. ^3^H-labeled cholesterol was purchased from PerkinElmer (Waltham, MA). Ac-LDL and human HDL were from Biomedical Technologies (Stoughton, MA).

### Western blotting

Macrophages were incubated in the absence or presence of 100 µg/ml ac-LDL for 48 h. The cell lysate was loaded onto 4–20% SDS-PAGE gels for electrophoresis. The protein was then transferred to nitrocellulose membranes (Amersham Biosciences, Pittsburgh, PA). Primary antibody and HRP-conjugated secondary antibody were used to detect target proteins. Signal was detected using an ECL kit (Amersham Biosciences). Rabbit anti-mouse ABCA1 and anti-mouse ABCG1 antibodies were from Novus Biologicals (Littleton, CO). Rabbit anti-β-actin antibody was from Sigma. Goat anti-rabbit secondary antibody was purchased from Millipore.

### Statistical analysis

Data are presented as the mean ± SD. Differences were compared with two-tailed Student’s *t*-test or one-way ANOVA using GraphPad Prism software (GraphPad Software Inc., San Diego, CA). P < 0.05 was considered statistically significant.

## Results

### MCPIP1 deficiency in bone marrow resulted in growth retardation and severe hematological disturbance

**Figure 1:**
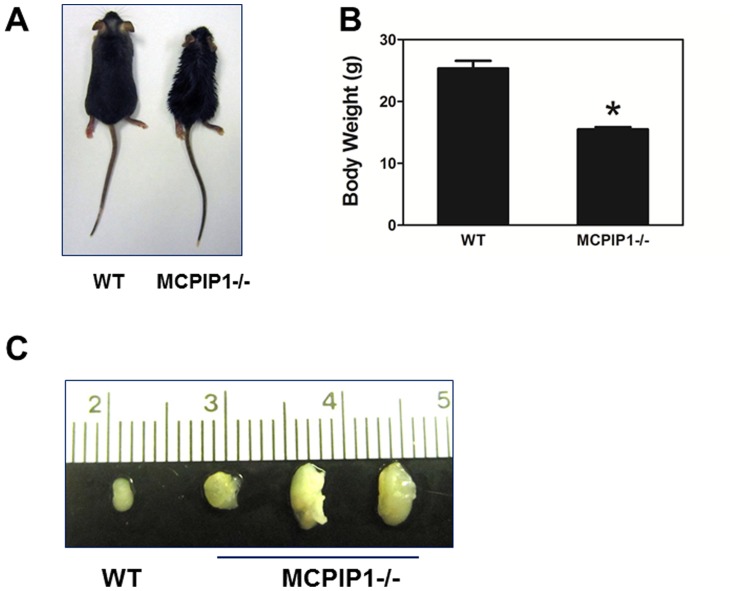
Bone marrow MCPIP1 deficiency resulted in growth retardation and lymphadenopathy. **A**. Representative images of LDLR−/− mice received bone marrow cells from WT (WT) or MCPIP1 deficient (MCPIP1−/−) mice. **B**. Body weights of mice. *p<0.01, n = 6 (WT) or 9 (MCPIP1−/−). **C**. Representative images of mouse lymph nodes.

**Figure 2:**
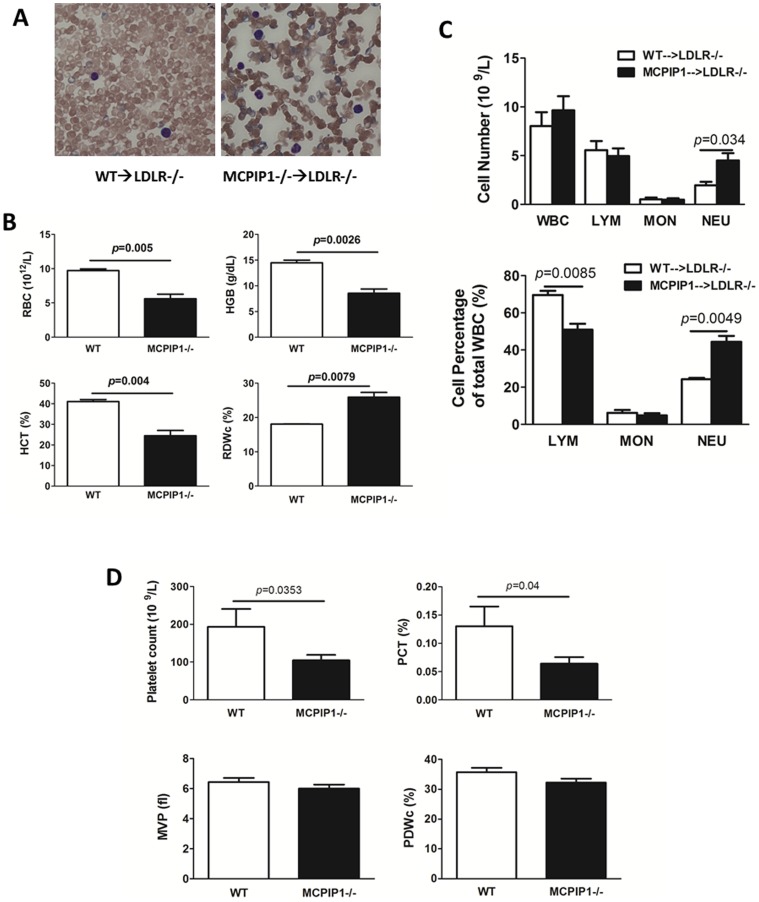
Bone marrow MCPIP1 deficiency caused hematological disturbance. **A**. Giemsa stained blood smears showed MCPIP1−/− bone marrow cell recipients contain dramatically reduced number of red blood cells. **B**. Vetscan HMT blood cell analysis showed reduced blood red cell (RBC) number, hemoglobin (HGB) concentration and hematocrit (HCT), as well as increased red cell distribution width (RDWc) which is calculated as Standard deviation ÷ mean cell volume x 100. **C**. Blood leukocyte profile showed increased number of neutrophils in MCPIP1−/− bone marrow cell recipients. **D.** Platelet related parameters of mice obtained by Vetscan HMT analysis. Parameters include total blood platelet count, platelet hematocrit (PCT), mean platelet volume (MVP), and platelet distribution width (PDWc) which is calculated using equation RDWc(%)  =  (Standard deviation ÷ mean cell volume) x 100. N = 6 (WT) or 9 (MCPIP1−/−).

We transplanted 5×10^6^ bone marrow cells from either WT or MCPIP1^−/−^ mice to lethally irradiated 6 wk old female LDLR^−/−^ mice. Four weeks following the bone marrow transplantation, the recipients were started on a western-type diet (WD). During the WD feeding period, we observed that even though the food intake was not different between the two groups and there was no obvious diarrhea (data not shown), the MCPIP1^−/−^ bone marrow recipients (hereafter referred as MCPIP1-BM mice) displayed a slower growth rate than WT bone marrow recipients (hereafter referred as WT-BM mice). After 7 wks on the WD, all MCPIP1-BM mice showed weakness and sickness symptoms and in fact one of them (10%) died. We therefore terminated the experiment, and sacrificed the remaining MCPIP1-BM mice and the WT-BM mice for analysis. MCPIP1-BM mice were much smaller and weighed significantly less than WT-BM mice (14.0±0.54 g vs 24.2±0.84 g, *p*<0.0001, **Figs 1A and 1B**). Similar to whole body knockout mice, MCPIP1-BM mice had significantly enlarged lymph nodes (**Fig 1C**). We and others previously reported that whole body MCPIP1 knockout mice displayed severe anemia and an abnormal blood cell profile[3], [4]. To examine if this phenotype was also transferred by bone marrow transplantation, we performed a detailed hematological analysis. Blood smear analysis showed that there were significantly fewer red blood cells in MCPIP1-BM mice than in WT-BM mice (**Fig 2A**). Vetscan HMT analysis indicated that red blood cell number, hemoglobin concentration and hematocrit were all significantly reduced in MCPIP1-BM mice, and the red cell distribution width was significantly increased (**Fig 2B**). Vetscan HMT analysis further showed that MCPIP1-BM mice had a slightly increased number of total white blood cells, a significant increase in neutrophil number as well as an increase in the percentage of lymphocytes and neutrophils compared to WT-BM mice (**Fig 2C**). Total blood platelet numbers and platelet hematocrit were significantly reduced in MCPIP1-BM mice compared to WT-BM mice; however, there were no differences in mean platelet volume or platelet distribution width between the groups (**Fig. 2D**). These data suggest that bone marrow transplantation transferred the disturbed hematopoietic phenotype.

### MCPIP1 deficiency in bone marrow caused immune organ disorganization and immune cell population alteration

**Figure 3:**
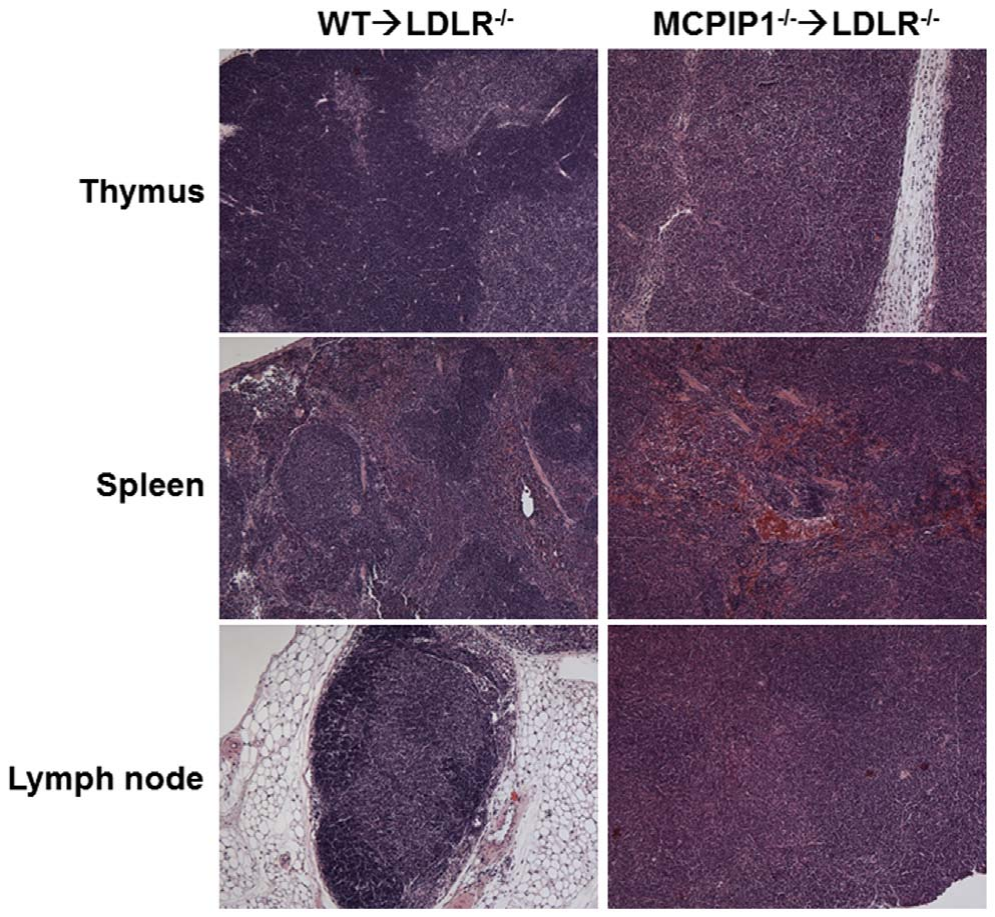
Bone marrow MCPIP1 deficiency resulted in disorganization of mouse immune organs and leukocyte profile alteration in the spleen. Light microscopic images of mouse thymus, lymph nodes and spleen which were stained with hematoxylin and eosin. Magnification: 4X.

**Figure 4:**
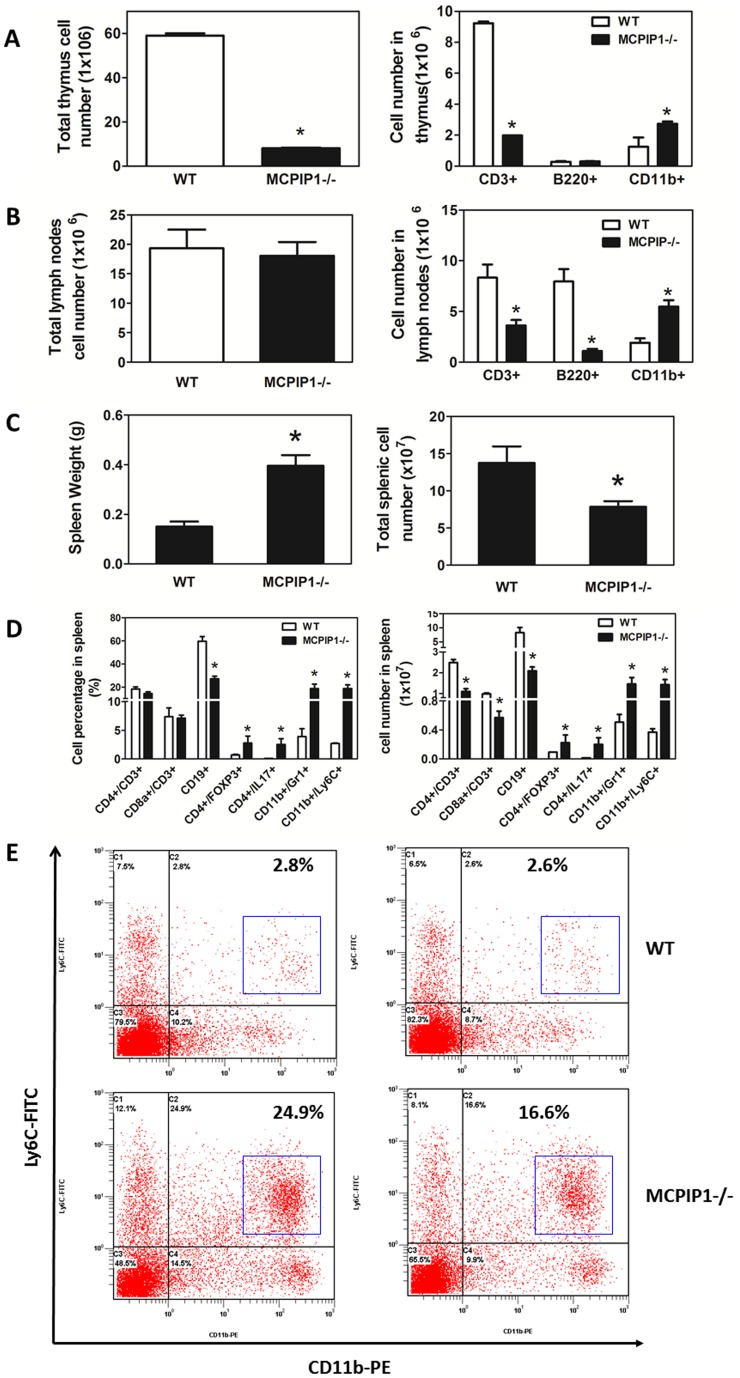
Bone marrow MCPIP1 deficiency altered leukocyte profile in immune organs. **A and B.** Total number and flow cytometric analysis of immune cells in mouse thymus **(A)** and lymph nodes **(B).** *p<0.05 vs WT group. **C.** Spleen weight and total spleen cell numbers of LDLR−/− mice received WT or MCPIP1−/− bone marrow cells. **D**. Leukocyte profile of splenocytes by flow cytometry. *p<0.01, n = 6 (WT) or 9 (MCPIP1−/−). **E.** Representative flow cytometric graphs of spleen CD11b+/Ly6C+ cells. Majority of the CD11b+/Ly6C+ cells are CD11b+/Ly6C^low^ (Blue box).

We and others have previously reported that MCPIP1 plays a central role in immune system development and function, and that MCPIP1 knockout mice have disorganized immune organs and impaired immune function [3], [4]. In the current study, we examined if bone marrow transplantation transferred the phenotype of MCPIP1^−/−^ mice to bone marrow recipients. The MCPIP1-BM mice had significantly enlarged lymph nodes and spleen compared to WT-BM mice, but their thymus sizes were similar. H & E staining of lymph organs showed striking abnormality in the thymus, spleen and lymph nodes (**Fig 3**). In the thymus, the cortex structure disappeared in MCPIP1-BM mice; the whole thymus contained only medulla-like structure. Total cell numbers in the thymus of MCPIP1-BM mice were significantly reduced (8.1±0.3×10^6^ cells vs 59.0±1.0×10^6^ cells in WT-BM mice), with significantly reduced number of CD3+ T cells but increased number of CD11b+ cells (**Fig 4A**). Although MCPIP1-BM mice had significantly enlarged lymph nodes, the cell numbers in lymph nodes were not different than WT-BM mice (**Fig 4B**). However, the lymph nodes of MCPIP1-BM mice had significantly fewer CD3+ T cells and B220+ B cells but significantly more CD11b+ cells compared to those of WT-BM mice (**Fig 4B**). The structure of the lymph nodes of MCPIP1-BM mice was also disorganized such that the lymph nodule was not distinguished (**Fig 3**). In the spleen of MCPIP1-BM mice, the boundary between the germinal center and the red pulp was blurred (**Fig 3**). Even though the spleens of MCPIP1-BM mice were two-fold the size of WT-BM mice, the cell numbers were only 2/3 of those of WT-BM mice (**Fig 4C**). The spleen serves as the reservoir of circulating leukocytes, thus we examined the leukocyte profile in the spleen using flow cytometry. First we detected the percentages of each leukocyte type based on cell surface markers, then calculated the absolute numbers of each cell type in the whole spleen of each mouse. We found that the spleens of MCPIP1-BM mice contained significantly fewer CD3+/CD4+ T cells, CD3+/CD8+ T cells and CD19+ B cells, however, they had significantly increased numbers of CD4+/FOXP3+ regulatory T cells, CD4+/IL17+ Th17 cells, CD11b+/Gr1+ myeloid-derived suppressor cells (MDSCs) and CD11b+/Ly6C+ monocytes (**Fig 4D**). The increased CD11b+/Ly6C+ cells were almost exclusively Ly6C^low^ cells (**Fig 4E**).

### MCPIP1 deficiency in bone marrow promoted systemic and multi-organ inflammation

**Figure 5:**
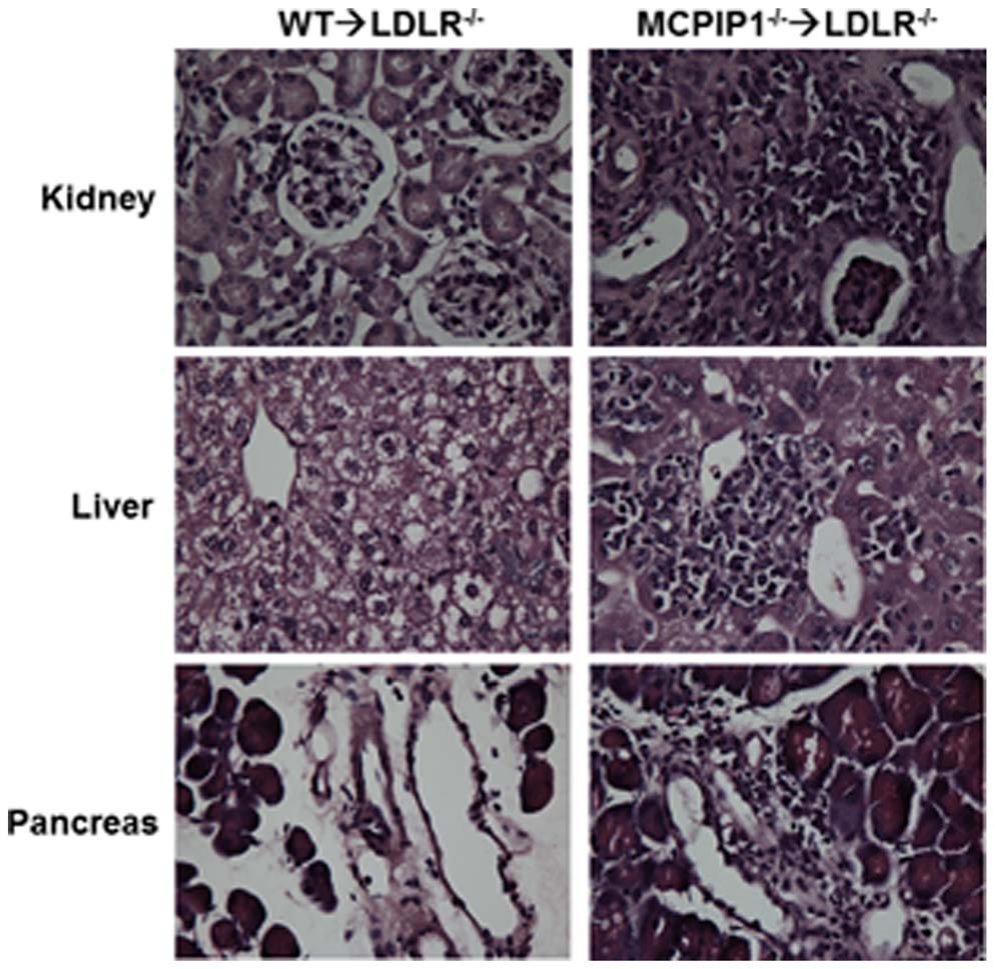
Bone marrow MCPIP1 deficiency caused multi-organ inflammation. Cryosections of kidney, liver and pancreas were stained with hematoxylin and eosin, and viewed with a Nikon Eclipse E600 microscope. Magnification: 40X

**Figure 6:**
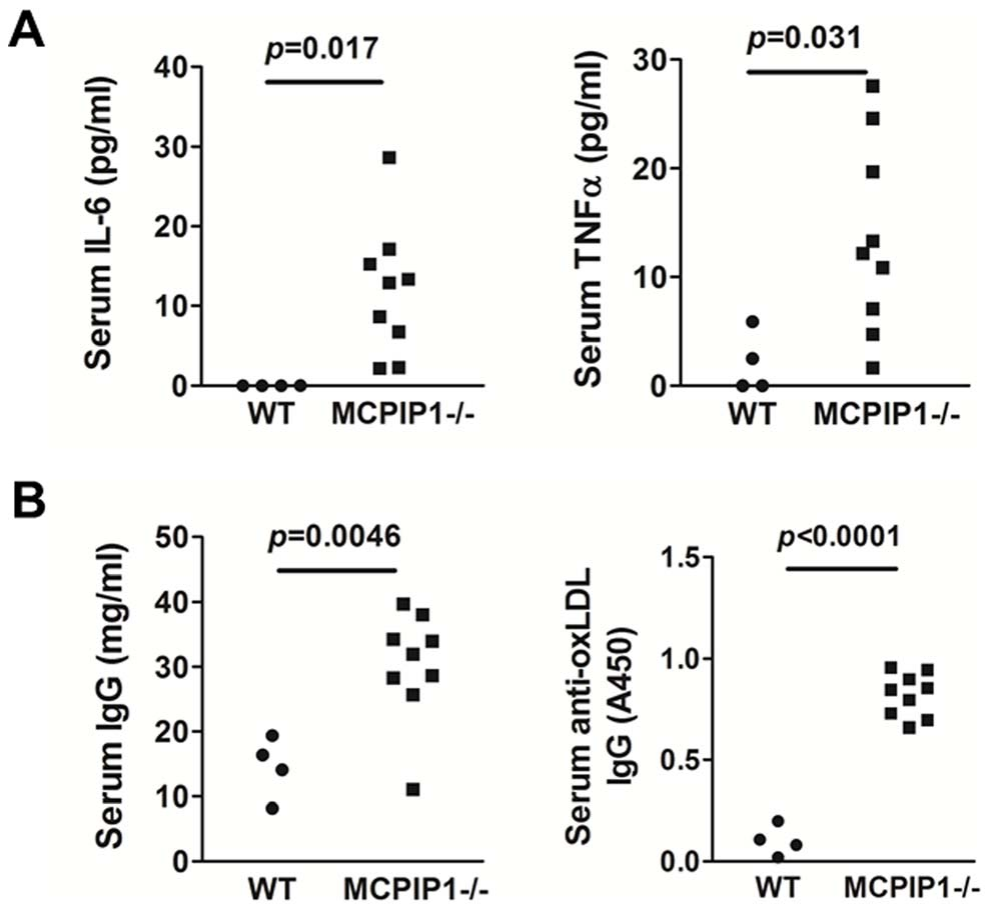
Bone marrow MCPIP1 deficiency increased mouse serum levels of proinflammatory cytokines and total and anti-oxLDL antibodies. **A.** Serum levels of pro-inflammatory cytokines TNFα and IL-6 were determined by ELISA. **B.** Serum levels of total IgG and anti-oxLDL IgG were determined by ELISA as described in Materials and Methods.

Whole body MCPIP1 knockout mice have been shown to display multi-organ inflammation. Therefore, we next examined if bone marrow deficiency of MCPIP1 results in a similar phenotype. We found that there were a large amount of inflammatory cell infiltration in many solid organs including kidney, liver and pancreas in MCPIP1-BM mice. In WT-BM mice, the microscopic appearances of these organs were mostly normal, except in the liver where most of the hepatocytes are foamy due to lipid accumulation (**Fig 5**). In MCPIP1-BM mouse kidney, cluster infiltration of inflammatory cells, mostly mononuclear cells, was found in the renal cortex predominantly near the arteries and veins. In the liver of MCPIP1-BM mice, the inflammatory cells accumulated around the central veins, also forming large clusters of cells. In the pancreas of MCPIP1-BM mice, many inflammatory cells infiltrated in the connective tissue septa between pancreatic lobules; most of the infiltrated inflammatory cells were mononuclear cells. We examined the proinflammatory cytokine levels in the serum of the mice using ELISA. We found that while the IL-6 and TNFα levels in the WT-BM mice were very low, even undetectable in some mice, the levels in MCPIP1-BM mice were significantly increased (**Fig 6A**). We also measured the total IgG and anti-oxidized LDL IgG in the mouse serum using ELISA, and found that MCPIP1-BM mice had significantly increased levels of both total IgG and anti-oxLDL IgG (**Fig 6B**). These data suggest that MCPIP1-BM had severe systemic and multi-organ inflammation.

### MCPIP1 deficiency in bone marrow slightly reduced serum cholesterol while dramatically diminished atherogenesis

**Figure 7:**
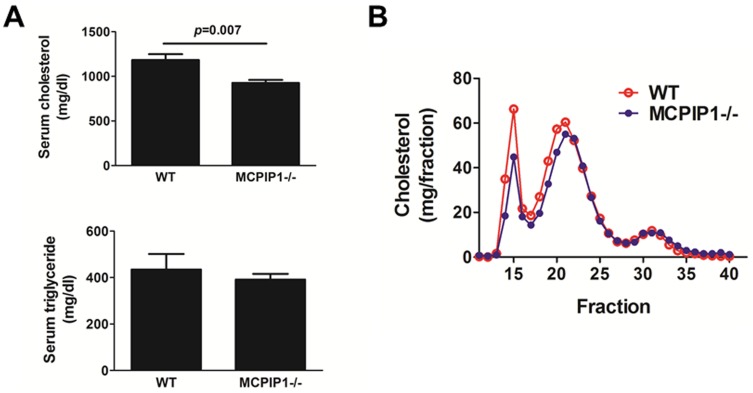
Bone marrow MCPIP1 deficiency slightly increased mouse serum total cholesterol levels. **A**. Serum total cholesterol and triglycerides. N = 6 (WT) or 9 (MCPIP1−/−). **B**. Serum lipoprotein profiles obtained by FPLC. Serum from three mice each group were pooled for FPLC analysis. Cholesterol and triglyceride concentrations of each fraction were measured by enzymatic colorimetric assays.

**Figure 8:**
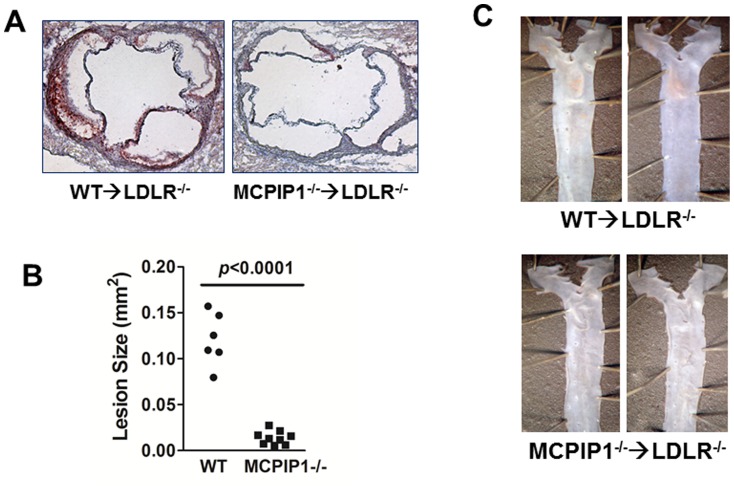
Bone marrow MCPIP1 deficiency dramatically reduced atherosclerotic lesion size in mouse aorta. **A**. Representative atherosclerosis lesions of aortic root. **B**. Quantification of aortic root atherosclerotic lesion size. **C**. Representative *en face* aorta atherosclerosis.

Even though the MCPIP1-BM mice displayed significant growth retardation, we did not find their intake of food to be less than WT-BM controls and we did not observe obvious diarrhea. However, the total serum cholesterol was slightly, but significantly, decreased in MCPIP1-BM mice compared to WT-BM mice (926±33 vs 1183±64 mg/dL, *p* = 0.007) (**Fig 7A**). There was no difference in total triglycerides between the two groups. FPLC analysis indicated that the serum cholesterol reduction was distributed in VLDL and LDL fractions, but not in HDL fractions, while the overall profiles were similar in two groups (**Fig 7B**). We measured the atherosclerosis lesions in the proximal aorta by H&E staining and found a significant reduction of lesions in MCPIP1-BM mice (∼10 fold, p<0.0001) (**Figs 8A and 8B**). Aorta *en face* oil-red O staining revealed that while WT-BM mice showed obvious atherosclerotic lesions at the aortic arch and thoracic aorta following 7 wk of WD feedings, MCPIP1-BM mice displayed essentially no atherosclerotic lesions in *en face* aorta (**Fig 8C**). Since there were essentially no lesions in MCPIP1-BM mice, we were not able to compare the lesion composition between the two groups of mice. Given that the serum cholesterol levels were lower in MCPIP1-BM mice, we took a closer look at the relationship between serum cholesterol levels and lesion size. We found that the WT-BM mouse with the lowest serum cholesterol (989.1 mg/dL) developed substantial lesion in the proximal aorta (0.109 mm^2^) whereas the two MCPIP-BM mice with highest serum cholesterol in the group (1038.4 and 934.8 mg/dL, respectively) developed very little lesion (0.021 and 0.016 mm^2^, respectively). Therefore, we believe that the slightly lower serum cholesterol levels could not sufficiently explain the significant reduction in lesion size in the MCPIP1-BM mice.

### MCPIP1 deficiency enhanced macrophage cholesterol efflux

**Figure 9:**
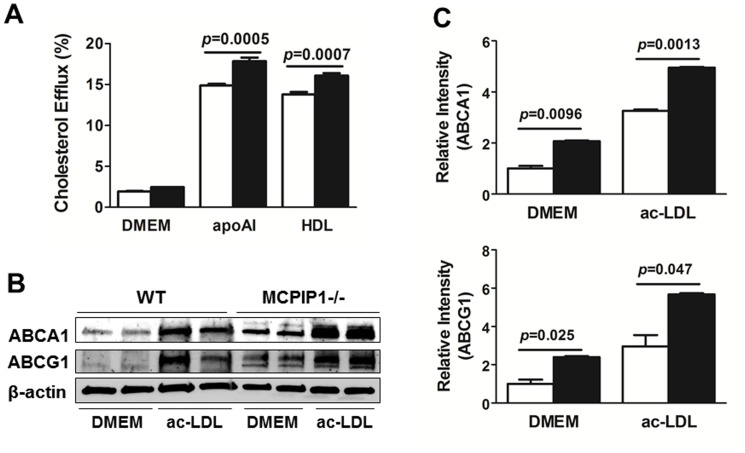
MCPIP1 deficiency increased macrophage cholesterol efflux. **A**. Bone marrow-derived macrophages were loaded with ac-LDL and cholesterol efflux to DMEM, apoAI and HDL was measured. Data were presented as mean ± SEM. N = 3 in each group. **B**. Bone marrow-derived macrophages were cultured in DMEM for 48 h with or without ac-LDL. Cell lysates were used for western blotting analysis for ABCA1 and ABCG1. Representative western blotting result was shown. **C**. Quantitative analysis of the western blots. Data were presented as mean ± SEM. N = 4 in each group. Open column: WT macrophages; Black column, MCPIP1−/− macrophages.

To further investigate the mechanism underlying the diminished atherogenesis in bone marrow MCPIP1 deficient mice, we examined the cholesterol efflux capability of bone marrow-derived macrophages (BMDMs) from WT and MCPIP1^−/−^ mice. BMDMs were loaded with ac-LDL for 48 h before efflux medium was applied to mediate cholesterol efflux to DMEM, apoAI (ABCA1-dependent) and HDL (ABCG1-dependent). **Figure 9A** showed that, compared with WT BMDMs, MCPIP1 deficient BMDMs displayed significantly enhanced cholesterol efflux to both apoAI (17.84±0.45% vs. 14.88±0.20%) and HDL (16.09±0.28% vs. 13.77±0.30%). In separate experiments, we performed western blots to examine the protein levels of ABCA1 and ABCG1 in the macrophages with or without ac-LDL loading. The data showed that, in both non-loaded cells and ac-LDL loaded BMDMs, MCPIP1 deficiency significantly increased protein levels of both ABCA1 and ABCG1 (**Figs. 9B and 9C**).

## Discussion

Given the anti-inflammatory role of macrophage-induced MCPIP1along with the profound hematological disturbance and immune dysfunction phenotype of MCPIP1 deficient mice, we aimed to investigate 1) if bone marrow deficiency of MCPIP1 can replicate the phenotype of whole body MCPIP1 knockout mice, and 2) if the inflammation resulting from bone marrow MCPIP1 deficiency translates into enhanced atherosclerosis, a chronic inflammatory disease in hyperlipidemic mice. There were two major findings in this study. First, transplantation of MCPIP1 deficient bone marrow sufficiently transferred all major phenotypes of MCPIP1 knockout mice to recipient mice, including growth retardation, shortened life-span, severe anemia, splenomegaly, lymphadenopathy, and multi-organ infiltration of inflammatory cells. Second, despite the systemic inflammatory phenotype, the LDLR^−/−^ mice that received MCPIP1^−/−^ bone marrow and consumed an atherogenic diet did not develop more atherosclerosis compared to the mice that received the wild type bone marrow cells; instead atherogenesis was essentially abolished in these mice.

The diseased phenotype of whole body MCPIP1 deficient mice has been described previously by our laboratory as well as others [3], [4]. Even though it has previously been noted that bone marrow can transfer the phenotype of MCPIP1 deficient phenotype [4], this current study is the first one to describe the phenotype of the chimeric mice in detail. While the bone marrow MCPIP1^−/−^ chimeric mice developed severe anemia as do MCPIP1^−/−^ mice, the chimeric mice did not have significantly increased white blood cells and platelets as MCPIP1^−/−^ mice, instead their platelet numbers were actually reduced compared to control mice. In addition, these mice had a reduced percentage of lymphocytes but an increased percentage and absolute number of neutrophils in the blood as measured by VetScan analysis. Blood smear Giemsa staining did not reveal a significant increase in plasma cells in the blood. While flow cytometrical analysis of splenocytes showed that the ratio of CD4+ to CD8+ T cells was not altered in bone marrow MCPIP1^−/−^ chimeric mice, similar to previous reports, the absolute numbers of both subsets of T cells was significantly reduced. We detected a significant increase in B cells, not only in the percentage but also in the absolute numbers. We also found that the percentages as well as absolute numbers of CD4+/FoxP3+ T_regs_ and CD4+/IL17+ Th17 cells were significantly increased in the chimeric mice. Interestingly, we found strikingly increased numbers of CD11b+/Gr1+ cells and CD11b+/Ly6c+ cells in the bone marrow MCPIP1−/− chimeric mice compared to the control mice, which was not previously reported in whole body MCPIP1^−/−^ mice. Given that the molecular and cellular mechanisms underlying the MCPIP1 deficiency-induced anemia and immune disturbance in the developmental stages are not clear, we will in the future use the bone marrow MCPIP1^−/−^ chimeric mice as a valuable animal model for this purpose by monitoring the hematopoietic cell differentiation and maturation over time following the bone marrow transplantation.

The most striking finding of this study is the paradoxical impact of bone marrow MCPIP1 deficiency on atherogenesis. Atherosclerosis is characterized by accumulation of lipids and inflammatory cells, mainly macrophages in the intima of arteries. Although the whole body MCPIP1 deficient mice display severe systemic and multi-organ inflammation, including accumulation of numerous inflammatory cells in the adventitia of the aorta, we did not find any atherosclerotic lesions in either the aortic root or the *en face* aorta (data not shown). This is consistent with the common concept that mice do not spontaneously develop atherosclerosis without hyperlipidemia. Given the short life span (most <12 weeks) of MCPIP1 deficient mice, it is not practical to study atherogenesis in MCPIP1^−/−^ mice even with high fat diet feedings. It also prevents the crossing of MCPIP1^−/−^ mice to either apoE^−/−^ or LDLR^−/−^ mice, two most commonly used mouse models for the study of atherosclerosis. Since bone marrow transplantation can effectively transfer the inflammatory phenotype resulting from MCPIP1 deficiency, this approach should provide insight into the role of MCPIP1 in atherogenesis. ApoE^−/−^ mice were not used because bone marrow cells expressing apoE will correct hyperlipidemia and essentially abolish atherogenesis[18]. Instead we used LDLR^−/−^ mice as bone marrow recipients; western diet feeding promotes atherogenesis in these mice and bone marrow deficiency of MCPIP1 may affect this process. We expected that bone marrow MCPIP1 deficiency would result in enhanced atherosclerosis in hyperlipidemic mice. The sickness and premature death of the bone marrow MCPIP1^−/−^ chimeric mice prevented us from feeding the mice for a longer duration; and we terminated the animal experiments after 7 wks on this diet (total 11 weeks after bone marrow transplantation). Surprisingly we found that atherogenesis was essentially abolished in the bone marrow of MCPIP1 deficient LDLR^−/−^ mice, while the control mice with WT bone marrow developed substantial atherosclerotic lesions in the proximal aorta, and initial lesions in the *en face* aorta.

We noticed that bone marrow MCPIP1 deficient mice had slightly, but significantly, decreased plasma total cholesterol levels compared to the WT bone marrow recipients. The slight decrease in plasma cholesterol mainly resided in VLDL and LDL fractions. While this reduction in plasma cholesterol levels would definitely have contributed to the reduced atherogenesis, we believe it is not sufficient to explain the dramatic diminishment of atherosclerosis in MCPIP1^−/−^ bone marrow recipients. For instance, the two bone marrow MCPIP1 deficient mice with the highest total plasma cholesterol level in the group (1038.4 mg/dl and 934.8 mg/dl respectively) developed 0.021 and 0.016 mm^2^ atherosclerotic lesions in the proximal aorta, about the average atherosclerosis as the other mice in this group whereas the mouse in the WT group with the lowest total plasma cholesterol (989.1 mg/dl) developed a 0.109 mm^2^ lesion, about the average lesion size of the WT group.

Besides the slight reduction of plasma cholesterol levels, the leukocyte profile of the mice may, at least in part, contribute to this phenomenon. Although there is strong evidence of systemic inflammation in the bone marrow MCPIP1^−/−^ mice, including inflammatory cell infiltration in multiple organs, increased pro-inflammatory and pro-atherogenic plasma cytokines (TNFα and IL-6), and increased total serum IgG and anti-oxidized LDL IgG, which are commonly believed to be pro-atherogenic [16], [19] but may also have potential protective effects against atherosclerosis [20], the leukocyte profile in the spleen is overall anti-atherogenic. The total numbers of CD4+ T cells and CD8+ T cells, generally considered pro-atherogenic [21], were reduced in bone marrow MCPIP1^−/−^ mice. The role of B cells in atherogenesis is more complicated; B1 have been shown to be athero-protective [22], [23], while B2 lymphocytes are pro-atherogenic [24]. The ratio of B1/B2 in the increased B cell population in bone marrow MCPIP1^−/−^ mice warrant further examination. CD4+/FoxP3+ regulatory T cells have been demonstrated to be anti-atherogenic [25], [26], and were significantly increased in bone marrow MCPIP1^−/−^ mice. But the number of CD4+/IL17+ Th17 cells, which are considered pro-atherogenic [27], [28], was also increased in bone marrow MCPIP1^−/−^ mice. Interestingly, CD11b+/Gr1+ cells, myeloid-derived suppressor cells (MDSCs), were significantly increased in bone marrow MCPIP1^−/−^ mice. There are currently no studies that have examined the role of MDSCs in atherogenesis, but given their inhibitory effects on T cell maturation and activation [29], [30], the increased number of MDSCs in bone marrow MCPIP1^−/−^ mice is likely to contribute to attenuation of atherogenesis. We also found that CD11b+/Ly6C+ cells, mostly of the Ly6C^low^ subset, were significantly increased in bone marrow MCPIP1^−/−^ mice. In hypercholesterolemic mice, the CD11b+/LyC^hi^ monocyte subset is believed to participate in atherogenesis, whereas CD11b+/LyC^low^ monocytes are associated with inflammation resolution [31]. Therefore, the increased CD11b+/Ly6C+, especially CD11b+/Ly6C^low^ monocytes may also contribute to reduced atherogenesis in the bone marrow MCPIP1^−/−^ mice. In summary, the leukocyte profile of spleen, which serves as a reservoir of circulating leukocytes, provides an overall anti-atherogenic merit. Nevertheless, the increasing pro-inflammatory cytokines, TNFα and IL-6, are clear evidence of an elevated systemic inflammatory status.

Since macrophage foam cell formation is an obligatory step in atherogenesis, impaired cholesterol efflux is a major contributory factor of macrophage cholesterol accumulation [32], we examined if MCPIP1 deficiency altered cholesterol efflux capability of cholesterol-loaded macrophages. While we did not find difference in ac-LDL uptake by WT and MCPIP1^−/−^ macrophages (data not shown), our data showed that MCPIP1^−/−^ macrophages displayed significantly enhanced capability to efflux cholesterol both to apoAI and to HDL, compared to WT macrophages. We further found this was due to increased ABCA1 and ABCG1 protein levels in MCPIP1^−/−^ macrophages. We believe that the enhanced cholesterol efflux capability of MCPIP1^−/−^ macrophages also contributed to the diminished atherogenesis in bone marrow MCPIP1 deficient mice.

A few conceptual scenarios can be raised based on the paradoxical findings from this study and we will test them in future studies. One, MCPIP1 may play diverse roles in the differentiation, maturation and activation in different immune cell sets and subsets. Under the influence of MCPIP1, some sets or subsets of immune cells may gain pro-atherogenic properties, while others may gain anti-atherogenic features. We are currently generating cell type-specific MCPIP1 knockout or transgenic mouse models to dissect the cell type-specific roles of MCPIP1 in the biology of T cells, B cells, monocytes/macrophages, and neutrophils. Two, the underlying molecular and cellular mechanisms of the inflammation in arterial wall and atherosclerosis and of the inflammation in other organs or diseases may be substantially different. The pro-inflammatory mechanisms responsible for the multi-organ inflammation in MCPIP1^−/−^ mice could indeed play a protective role in atherosclerotic inflammation in the arterial wall. For example, whereas MDSCs are an undesirable component of the inflammation in cancer microenvironment [33] and a normal component of the inflammatory responses in sepsis and trauma [34], they may play a protective role in atherogenesis. We will next analyze the nature of the inflammatory response in solid organs versus arterial wall using our current mouse model and the cell type-specific MCPIP1 knockout and transgenic mouse models. Third, the capability of macrophages to maintain cholesterol homeostasis may be more dominant than the overall systemic inflammatory stress in determining the progress of atherosclerosis. If macrophages maintain the ability to prevent cholesterol accumulation and foam cell formation, systemic inflammation does not significantly promote atherogenesis.

In conclusion, this study shows that bone marrow deficiency of MCPIP1 in hyperlipidemic mice promoted systemic and multi-organ inflammation but diminished atherogenesis. While the slightly reduced plasma cholesterol levels, the potentially anti-atherogenic leukocyte profile, and enhanced macrophage cholesterol efflux capability may collectively explain the reduction in atherogenesis in bone marrow MCPIP1^−/−^ mice, future studies are warrant to further determine the role of systemic inflammation and leukocyte cell type-specific MCPIP1 expression in atherogenesis.
